# Mutation in the *STXBP1* Gene Associated with Early Onset West Syndrome: A Case Report and Literature Review

**DOI:** 10.3390/pediatric14040046

**Published:** 2022-09-20

**Authors:** Kanako Takeda, Yusaku Miyamoto, Hisako Yamamoto, Toshiyuki Iwasaki, Noriko Sumitomo, Eri Takeshita, Atsushi Ishii, Shinichi Hirose, Naoki Shimizu

**Affiliations:** 1 Department of Pediatrics, Kawasaki Municipal Tama Hospital, 1-30-37 Shukugawara, Tama-ku, Kawasaki City 214-8525, Kanagawa, Japan; 2 Department of Pediatrics, St. Marianna University School of Medicine, 2-16-1 Sugao, Miyamae-ku, Kawasaki City 216-8511, Kanagawa, Japan; 3 Department of Child Neurology, National Center of Neurology and Psychiatry, 4-1-1 Ogawahigashi-machi, Kodaira City 187-8511, Tokyo, Japan; 4 Department of Pediatrics, Fukuoka Sanno Hospital, 3-6-45 Momochihama Sawara-ku, Fukuoka-City 814-0001, Fukuoka, Japan; 5 Department of Pediatrics, Fukuoka University School of Medicine Comprehensive Medical Research Center, 45-1 7-Chome Nanakuma Jonan-Ku, Fukuoka-City 814-0180, Fukuoka, Japan

**Keywords:** developmental and epileptic encephalopathies, ACTH therapy, severe intellectual disability

## Abstract

Syntaxin-binding protein1 (*STXBP1*) is a member of the Sec1/Munc18-1 protein family, which comprises important regulators of the secretory and synaptic vesicle fusion machinery underlying hormonal and neuronal transmission, respectively. *STXBP1* pathogenic variants are associated with multiple neurological disorders. Herein, we present the case of a Japanese girl with a mutation in the *STXBP1* gene, who was born at 40 weeks without neonatal asphyxia. At 15 days old, she developed epilepsy and generalized seizures. Around 88 days old, she presented with a series of nodding spasms, with the seizure frequency gradually increasing. Interictal EEG indicated hypsarrhythmia and she presented with developmental regression. At 1.5 years old, genetic testing was performed and mutational analysis revealed an *STXBP1* gene mutation (c.875G > A: p.Arg292His). Accordingly, she was diagnosed with developmental and epileptic encephalopathy, presenting West syndrome’s clinical characteristics caused by the *STXBP1* gene mutation. Although drug treatment has reduced the frequency of epileptic seizures, her development has remained regressive. The relationship between the location and type of genetic abnormality and the phenotype remains unclear. Future studies should investigate the genotype–phenotype correlation and the underlying pathophysiology to elucidate the causal relationships among the multiple phenotype-determining factors.

## 1. Introduction

When determining the etiology of early-onset infantile seizures following neonatal seizures, genetic, congenital metabolic, and structural brain abnormalities as well as a hypoxic–ischemic state should be mainly considered. The major neonatal-onset epilepsy syndromes are self-limited neonatal epilepsy and early infantile developmental and epileptic encephalopathy (previously early myoclonic epilepsy [EME] and early infantile epileptic encephalopathy [EIEE]) [1]. Some neonatal seizures may present with conditions that are atypical of the classic EIEE or EME, which, due to repeated seizures, affect interictal brain function, leading to developmental delay and cognitive–motor function. This condition was reworded as developmental epileptic encephalopathy (DEE) in the 2017 revision of the International League Against Epilepsy [2]. More than 60 causative genes for DEE have been identified to date, including the syntaxin-binding protein 1 *(STXBP1*) gene.

STXBP1, also known as Munc-18-1, is a member of the Sec1/Munc18-1 protein family, which comprises important regulators of the secretory and synaptic vesicle fusion machinery involved in the hormonal and neuronal transmission, respectively [3]. Pathogenic *STXBP1* variants usually impair neurotransmitter release, specifically in GABAergic interneurons, which causes uncontrolled excitatory neuron firing [4]. Moreover, they are associated with developmental delay, cognitive dysfunction, intellectual disability, and epilepsy [3]. *STXBP1* encephalopathy with epilepsy is one type of DEE and is qualified by early-onset encephalopathy with epilepsy. Epilepsy syndromes include infantile spasms (IS), EIEE, Lennox–Gaustaut syndrome, Dravet syndrome (not *SCN1A*-related), classic Rett syndrome (not MECP2-related), and atypical Rett syndrome (not *CDKL5*-related) [4].

Patients with IS typically exhibit epileptic spasms along with the EEG pattern known as hypsarrhythmia. West syndrome is classified as a subtype of IS and is the most commonly described subtype, appearing in approximately 90% of instances of IS. It includes the combination of epileptic spasm, hypsarrhythmia, and developmental arrest or regression [5]. Over 130 other genes, in addition to *STXBP1*, have also been linked to IS. Examples include *ARX*, *CDKL5*, *PAFAH1B1/LIS1*, *DC*, and *KCNQ2* [6]. Here, we present a case of a patient with an *STXBP1* mutation who was clinically diagnosed with West syndrome.

## 2. Case Presentation

**Figure 1:**
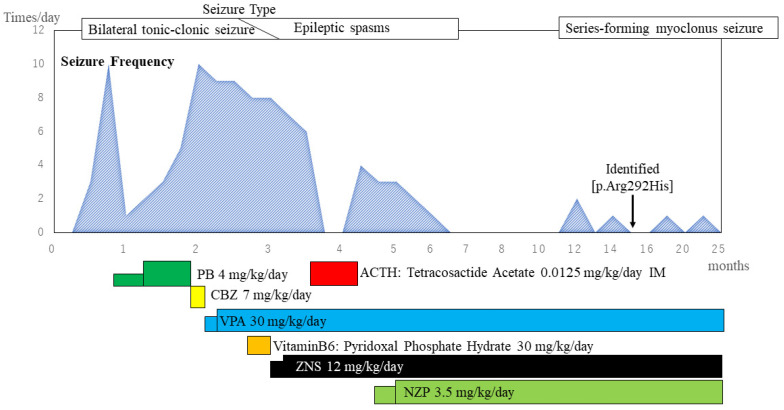
Clinical course of the treatment. Seizure frequency and ASM are shown in this figure. All ASMs except ATCH were administered orally. The type of seizure was initially bilateral tonic–clonic seizure, but changed to epileptic spasm from around 88 days old. Blood levels of the main ASM were VPA: 86.1 µg/mL, ZNS 18.1 ng/mL, and NZP 0.13 µg/mL at the mean trough values in maximum dosage. Abbreviations: ASM, antiseizure medication; PB, phenobarbital; CBZ, carbamazepine; VPA, valproic acid; ZNS, zonisamide; NZP, nitrazepam; ACTH, adrenocorticotropic hormone; PO, by mouth (orally); IM, intramuscular.

Our patient was a girl who was 40 weeks in utero and born through natural vaginal delivery as the first child of a Japanese couple. Her birth weight, height, and head circumference were 3244 g (+0.67 standard deviation [SD]), 46.8 cm (−0.77 SD), and normal, respectively. Neonatal asphyxia was not observed at birth. Her family history lacked evidence regarding consanguineous marriage, epilepsy, or neuromuscular disease. The clinical course of the patient is shown in Figure 1. At 15 days old, she presented with bilateral tonic–clonic seizures that lasted for a few minutes, which started in the left or right upper extremity. The seizure frequency increased to approximately 10 per day; the patient was admitted to the hospital by her previous doctor. Interictal electroencephalography (EEG) revealed multifocal spontaneous spikes. The EEG showed isolated spike waves in the right frontal, central, and occipital regions that did not spread widely. It was not a suppression-burst or hypsarrhythmia pattern. General blood tests, plasma amino acid analysis, general spinal fluid tests, spinal fluid virus isolation, and urinary organic acid analysis revealed no abnormalities. Brain magnetic resonance imaging (MRI) revealed no obvious epilepsy-inducing structural abnormalities. She was started on phenobarbital for neonatal-onset epilepsy of unknown classification; however, she showed no improvement. She was referred to our hospital at the age of 75 days.

**Figure 2:**
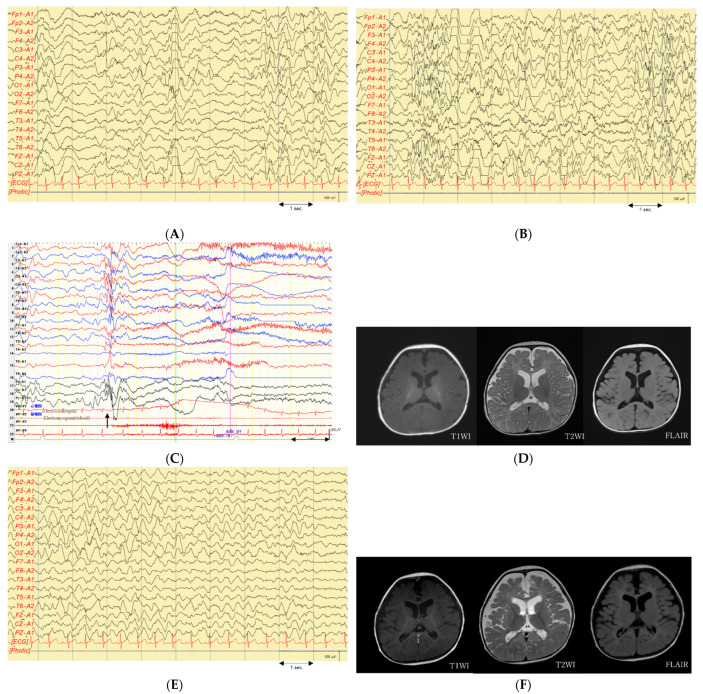
EEGs and brain MRIs of the patient. (**A**) Interictal EEG at 2 months of age showing localized spike waves and spike–slow wave complexes in the right frontal, central, and occipital regions, which further broadened. Additionally, a left central spike wave was observed; (**B**) Interictal EEG at 3 months of age showing frequent irregular high-amplitude spikes and slow waves without left–right synchrony during sleep and wakefulness, indicative of hypsarrhythmia; (**C**) Ictal EEG at 3 months of age revealing spasms coinciding with irregular poly-spike waves followed by high-amplitude slow-wave complexes (arrowed area) and desynchronization; (**D**) Brain MRI at 3 months of age shows no obvious structural abnormalities that could cause seizures; (**E**) Interictal EEG at 11 months of age revealing slow waves at 3–4 Hz that were continuously observed on both sides as background waves without hypsarrhythmia; (**F**) Brain MRI at 1.4 years of age showing diffuse cerebral atrophy and corpus callosum thinning; however, there were no obvious abnormal structures.

Physical examination and general neurological findings revealed no obvious facial abnormalities or deformities. Eye-tracking and smiling when someone touched or held her were appropriate for infant age. Interictal EEG revealed normal sleep waves; however, there were frequent localized spike waves and spike–slow wave complexes (Figure 2A). A tentative diagnosis of symptomatic localization-related epilepsy was made, and treatment was initiated. She continued to experience seizures approximately 10 times a day; therefore, phenobarbital was discontinued and carbamazepine was started at 78 days old. However, it was discontinued after a few days due to an increase in seizure frequency. Oral sodium valproate and zonisamide were initiated at 81 and 88 days old, respectively. Vitamin B6 was administered; however, it was discontinued after having no effect. After starting sodium valproate and zonisamide, there was a gradual decrease in the seizure frequency; however, even at the maximum dose, seizures were still observed 3–4 times a day. Initially, the seizures were generalized tonic convulsions starting from the left upper extremity. However, around the age of 88 days, she displayed a series of nodding spasms. After adjustment of medications, oral intake was decreased, and tube feeding was initiated. At 108 days old, the patient underwent examination for primary disease according to the Department of Child Neurology, National Center of Neurology and Psychiatry. Long-term EEG monitoring revealed frequent irregular high-amplitude spikes and slow waves without left-right synchrony during sleep and wakefulness, which indicated a hypsarrhythmia (Figure 2B). Ictal EEG revealed a fast-wave rhythm and positive slow waves preceding the spasm (Figure 2C). Eye tracking and chuckling during the early disease stage were no longer observed. Further, she was considered to be developmentally regressing and was diagnosed with West syndrome. Brain MRI revealed no structural abnormalities presenting as obvious epileptic foci, including focal cortical dysplasia (Figure 2D). The patient was started on adrenocorticotropic hormone (ACTH) therapy at 114 days old, which was administered daily for two weeks.

The seizures disappeared the day after starting ACTH therapy. Recurrent spasms were observed 8 days after initiating ACTH therapy. Subsequently, sodium valproate and zonisamide were continued; however, there was no change in seizure frequency. Nitrazepam was started at the age of 4 months, which decreased the seizure frequency to 1–2 per day. EEG revealed multifocal spikes; however, the typical hypsarrhythmia disappeared and there was improved background activity (Figure 2E). Cerebral cortical atrophy occurred as a side effect of ACTH therapy. Development remained regressive, without evidence of head control, eye tracking, or chuckling. Due to aspiration pneumonia, nutrition was maintained by tube feeding. The patient was discharged from the hospital at 6 months old. After 7 months old, the patient lacked obvious clinical seizures. At 10 months old, genetic testing for West syndrome was performed. Subsequently, she was hospitalized for a urinary tract infection and pneumonia at 10 months and 1.3 years old, respectively.

At approximately 1.2 years old, she began experiencing a series of seizures involving the twitching of her right leg during sleep for 2–3 min at a time. Ictal EEG revealed series-forming myoclonus seizures. Interictal EEG showed multifocal spikes and slow waves at sleep onset without hypsarrhythmia. Moreover, background activity during wakefulness revealed continuous slow waves. Brain MRI showed diffuse cerebral atrophy and corpus callosum thinning; however, there were no obvious abnormal structures (Figure 2F). Despite the observation of a new seizure type, three antiepileptic drugs were administered at extreme doses and no indication for active surgery was indicated.

**Figure 3:**
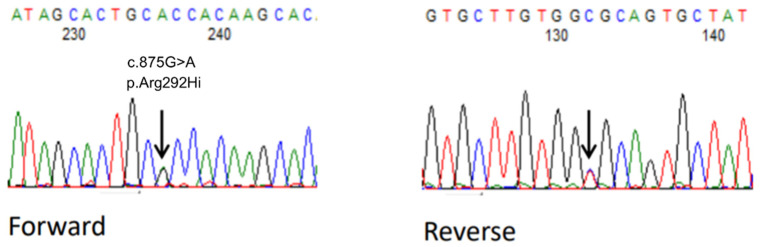
Monoallelic gene mutation c.875G > A [p.Arg292His] is revealed both in the forward and reverse sequences.

At 1.5 years old, she underwent further genetic testing. Target capture sequencing was performed to screen 114 epilepsy-related genes in the proband of the Department of Pediatrics at Fukuoka. We identified a missense variant (RefSeq accession number NM_003165: c.875G > A [p.Arg292His] available at https://www.ncbi.nlm.nih.gov/refseq/ (accessed on 27 July 2022)) in the *STXBP1* gene. Sanger sequencing confirmed the variant in the proband (Figure 3). A search for this variant in her parents through Sanger sequencing revealed that the variant was de novo. Finally, she was diagnosed with developmental and epileptic encephalopathies exhibiting clinical characteristics of West syndrome due to *STXBP1* gene mutation (c.875G > A [p.Arg292His], previously reported in ClinVar [rs796053361] as a pathogenic variant). Other unidentified variants in addition to *STXBP1*-p.Arg292His may have contributed to her clinical phenotype; since she did not undergo whole genome or whole exome sequencing, we continue to investigate this possibility.

The patient is currently 2.1 years old. Although the frequency of epileptic seizures has decreased, her development remains regressive, and she has a severe intellectual disability. At the time of writing, apart from the neurological phenotype, the patient had no other symptoms, such as metabolic disease, primary immunodeficiency, or hyperinflammatory condition. She continues to receive medical treatment. Should the seizures worsen in the future, we will review the choice of drug according to the seizure type and consider palliative epilepsy surgical treatment to reduce seizure frequency.

## 3. Discussion

This report describes a patient with West syndrome associated with genetic etiology related to an *STXBP1* gene mutation. West syndrome mostly occurs in the first year of life and presents as spasms with characteristic EEG changes known as hypsarrhythmia; moreover, it is strongly associated with developmental delay or regression [6,7]. *STXBP1* encephalopathy is a type of West syndrome that causes encephalopathy. It is characterized by early-onset encephalopathy, with about half of cases presenting seizures in the neonatal period. Frequent seizure types include epileptic spasms, focal seizures, and tonic seizures. Epileptic spasms frequently occur at some stage of the disease course (65.3%) [8]. In addition to epilepsy, a pathogenic de novo *STXBP1* gene mutation causes various neurological deficits, including severe early-onset forms of intellectual disability, autism, and movement disorders [3].

The patient experienced bilateral tonic–clonic seizures and had multifocal abnormalities on the EEG from the beginning of her epilepsy in her neonatal period, but not a suppression-burst pattern. Consequently, a diagnosis of neither conventional EIEE nor EME could be established, and the patient was first considered to be in a condition of DEE with no specific syndrome diagnosis and was followed up. Subsequently, the seizure type and EEG findings changed, developmental regression was observed, and the condition was diagnosed as West syndrome within the concept of DEE. Although many reports describe that the clinical spectrum of *STXBP1* mutations may be broader and there are differences between reports on the early features of onset, the presence of EEG suppression-burst patterns and epileptic spasms may better characterize *STXBP1* encephalopathy. According to a study by Saitsu et al., *STXBP1* encephalopathy first manifests as a range of seizure forms; however, 22% of patients also experienced epileptic spasms [9]. In a report of 24 cases of epileptic patients with *STXBP1* mutations, epileptic spasms were discovered in 78% of cases as the initial seizure. A hypsarrhythmic pattern was already evident from the initial EEG in one child at 2 months old, and multifocal asynchronous spikes and slow waves without discontinuity were seen in 78% and 27% of patients, respectively [10]. Even when there is not a noticeable early EEG suppression burst pattern, as there is in the current example, the likelihood of *STXBP1* mutation should be considered. Although EIEE is the most common epileptic syndrome phenotype in *STXBP1* encephalopathy (53%), a transition from EIEE to West syndrome was reported in 78% of patients (7 of 9 patients with EIEE) after a few to several months [9]. One of the features of this disease is that the type of seizure changes, as does the diagnosis. A previous study reported that the seizure types included bilateral tonic asymmetric, clonic, epileptic spasms, and bilateral axial myoclonic seizures, and that 60% of affected individuals had more than one seizure type during their lifetime [10,11]. In our case, three seizure types were identified during the patient’s clinical course, which is regarded as a manifestation of these symptoms.

In terms of neurodevelopment, the patient had seizures from the neonatal period; however, at the time of referral to our hospital at 2 months old, she was developing eye tracking and smiling when someone touched or held her. However, over the next three weeks, the seizure type changed to epileptic spasm, and the eye tracking and smiling disappeared, indicating a regression in neurodevelopment. Thereafter, she was unable to hold her head upright and was in the most severe state of psychomotor retardation. Previous reports indicate that 90% of patients with *STXBP1* gene mutations have severe intellectual disability, and autism or autistic features have been reported in 31% of these patients [8]. Existing case series suggest that there is considerable variability in the types and severity of neurodevelopmental problems associated with *STXBP1* variants, and this study revealed that, compared to the other intellectual disability group with pathogenic mutations in other intellectual disability-associated genes, the *STXBP1* group had more severe global adaptive impairments, fine motor difficulties, and hyperactivity [12]. In our case, West syndrome and the most severe psychomotor retardation were present; therefore, the severe degree of developmental delay may represent a feature of *STXBP1* encephalopathy.

With regard to the treatment of epilepsy induced by *STXBP1* encephalopathy, about 25% of patients with *STXBP1* encephalopathy were resistant to antiseizure medication (ASM) and more than 20% of patients with the condition required the use of two or more ASMs together [13]. The ketogenic diet was attempted to treat seizures in about 1% of patients with *STXBP1* encephalopathy, and response to the diet was either minimal or nonexistent [14,15]. The chosen course of treatment for epilepsy was surgery, such as corpus callosotomy [16] and resection of the patient’s affected cortex if they had focal cortical dysplasia [14].

In our case, there was no apparent effect of ACTH treatment. Liu et al. reviewed 15 patients with epilepsy associated with *STXBP1* gene abnormalities who were treated with ACTH and the seizures disappeared in 9 patients, although all these patients had severe psychomotor retardation [17]. Xian et al. conducted a comparative effectiveness assessment of ASM and treatment responses in *STXBP1*-related disorders in 534 individuals and ACTH therapy was more likely to reduce seizure frequency than to exacerbate symptoms or have no effect [18]. However, only partial and transient responses were reported in other studies [9,16]. The efficacy of ACTH therapy requires additional exploration; it may lessen seizures but has not been proven to enhance developmental prognosis. In the case series presented by Mihl et al., seizures disappeared in five patients between 6 and 18 months; however, severe psychomotor retardation persisted in all cases [11]. Moreover, *STXBP1* encephalopathy may be characterized by a lack of improvement in developmental outcomes after seizures have subsided, and it is necessary to explain to patients and their families that anti-epileptic drug therapy, such as ACTH therapy has significant side effects and cannot be expected to improve developmental outcomes. Thus, patients and/or their families should carefully consider whether it should be implemented.

The clinical course of *STXBP1*-related encephalopathy has not yet been established, although several case reports and case series have been published. The clinical features of STXBP1-related encephalopathy may be clarified by accumulating more cases for further studies.

Our patient carries the c.875G > A (p.Arg292His) missense mutation in the *STXBP1* gene, which has been previously reported in six patients (Table 1) [6,8,10,11,12,13,14,19,20,21,22,23], all of whom presented with epilepsy. Regarding the age at onset, four and two patients showed neonatal and childhood-onset, respectively. Six of the seven patients presented with developmental and epileptic encephalopathy; however, there were varying seizure types and EEG findings. The most unique case is Pt. 42, which was reported by Stamberger et al. [8]. Among these cases, there were clear differences in the disease course and seizure types. Although all cases showed global developmental delay, there were various phenotypic differences. Studies have reported a heterozygous loss of *STXBP1* function, followed by reduced expression, which causes impaired synaptic transmission. Although there is no obvious domain selectivity in missense mutations, most *STXBP1* mutations destabilize the original folding protein state, which increases the protein’s susceptibility to misfolding, aggregation, and degradation [3,24,25]. Moreover, recent reports indicate that *STXBP1* regulates the self-renewing aggregation of α-synuclein, which is a presynaptic protein involved in various neurodegenerative diseases; further, missense mutations co-aggregate with both wild-type *STXBP1* and α-synuclein [26]. *STXBP1* gene mutations or haploinsufficiency can cause pathogenic gain-of-function phenotypes by disrupting the native *STXBP1* function and reducing chaperone activity of α-synuclein, which causes delayed brain development and aggregation-induced neurodegeneration [3]. Given the phenotype diversity, various factors, such as other genetic and environmental factors, may be involved in determining the final phenotype. It is conceivable that these causes may have phenotypic differences.

## 4. Conclusions

Further research is required to investigate the genotype–phenotype correlation and the underlying pathophysiology, which may elucidate the causal relationships among the multiple phenotype-determining factors.

## Figures and Tables

**Table 1:** Information on patients with a previously reported *STXBP1* gene c.875G > A [p.Arg292His] missense mutation.

	Stamberger H [6]	Li.T [10]	Zhang Q [11]	Helbig KL [12]	Trump N [13]	Michaud JL [14]	Our patient
	(Pt. 42)	(Pt. 8)	(Pt. 123)	(Pt. 77)	(Pt. 64)	(Pt. 9)	
Sex/age at study	F	M/2 mo 3 d	F/0.8 mo	NR	M	M	F/2 Y
Age at onset	8 Y	15 d	3 d	Childhood	NR	7 d	15 d
Seizure semiology:initial	Focal behavior arrest	Eye blinking, lip smacking	NR	Focal Seizures	NR	Focal Seizures Epileptic spasms	FBTCS, FMS, Epileptic spasms, Myoclonic
Development prior to Seizure onset	Global developmental delay, severe ID						
EEG at onset	Sharp waves and poly spikes waves R central temporal regions (8 Y)	Burst-suppression during sleep, multifocal epileptic activity during awake status (2 mo)	NR	NR	Burst-suppression	Multifocal spikes	Multifocal spikes
Response to initial treatment	Oxcarbazepine: seizure free	No effect to PB	Refractory to AEDs	NR	NR	Refractory to AEDs (tried 7 drugs)	No effect to PB
MRI findings (age)	Normal (5.3 Y)	Normal (2 mo)	NR	NR	NR	Atrophy, Basal ganglia hyperintensities	Normal (3 mo)
Evolution of AEDs and other regimens	Not change	TPM, LEV, NZP, and Dex, MP, PRD	NR	NR	NR	NR	CBZ, Vitamin B6, VPA, ZNS, NZP
Clinical diagnosis	Non-convulsive status epilepticus	Ohtahara syndrome with evolution to West syndrome	Ohtahara syndrome	Early infantile epileptic encephalopathy	Early infantile epileptic encephalopathy	West syndrome	West syndrome
Current treatment	Oxcarbazepine	LEV, NZP	NR	NR	NR	NR	VPA, ZNS, NZP
Current status	Seizure free	Seizure free	Seizure not free	NR	NR	NR	Seizure not free
Clinical examination at the end of follow up	9 Y. Non ambulatory, No speech, Truncal and limb ataxia, Hyperreflexia hypotonia, Pubertas praecox	1 y 3 mo. Poor head control, unable to sit independently, poor social contact	Global developmental delay	Global developmental delay	Severe Global developmental delay	Global developmental delay, moderate ID from infancy	2 y 1 mo. Global developmental delay

Abbreviations: MP, methylprednisolone; DEX, dexamethasone; NZP, nitrazepam; PB, phenobarbital; PRD, prednisone; TPM, topiramate; VGB, vigabatrin; VPA, valproic acid; ZNS, zonisamide; AED, antiepileptic drug; NR, not reported; ID, intellectual disability; FBTCS, focal to bilateral tonic–clonic seizure; FMS, focal motor seizure.

## Data Availability

Not applicable.
